# SurvNet: A low-complexity convolutional neural network for survival time classification of patients with glioblastoma

**DOI:** 10.1016/j.heliyon.2024.e32870

**Published:** 2024-06-12

**Authors:** Qiyuan Lyu, Mario Parreno-Centeno, João Paulo Papa, Esin Öztürk-Isik, Thomas C. Booth, Fumie Costen

**Affiliations:** a Electrical and Electronic Engineering Department, The University of Manchester, Oxford Rd, Manchester M13 9PL, United Kingdom; b King's College London, Strand, London WC2R 2LS, United Kingdom; c School of Sciences, São Paulo State University, Brazil; d Institute of Biomedical Engineering, Bogazici University, Istanbul, Turkey; e Biomedical Engineering and Imaging Sciences, St. Thomas' Hospital, King's College London, London, United Kingdom; f Department of Neuroradiology, King's College Hospital NHS Foundation Trust, London, United Kingdom; g Image Processing Research Team, Centre for Advanced Photonics, RIKEN, Saitama, Japan

**Keywords:** Brain tumor glioma glioblastoma, Overall survival classification deep learning, Magnetic resonance imaging convolutional neural network

## Abstract

**Background and objective:**

Malignant primary brain tumors cause the greatest number of years of life lost than any other cancer. Grade 4 glioma is particularly devastating: The median survival without any treatment is less than six months and with standard-of-care treatment is only 14.6 months. Accurate identification of the overall survival time of patients with brain tumors is of profound importance in many clinical applications. Automated image analytics with magnetic resonance imaging (MRI) can provide insights into the prognosis of patients with brain tumors.

**Methods:**

In this paper, We propose SurvNet, a low-complexity deep learning architecture based on the convolutional neural network to classify the overall survival time of patients with brain tumors into long-time and short-time survival cohorts. Through the incorporation of diverse MRI modalities as inputs, we facilitate deep feature extraction at various anatomical sites, thereby augmenting the precision of predictive modeling. We compare SurvNet with the Inception V3, VGG 16 and ensemble CNN models on pre-operative magnetic resonance image datasets. We also analyzed the effect of segmented brain tumors and training data on the system performance.

**Results:**

Several measures, such as accuracy, precision, and recall, are calculated to examine the perfor-mance of SurvNet on three-fold cross-validation. SurvNet with T1 MRI modality achieved a 62.7 % accuracy, compared with 52.9 % accuracy of the Inception V3 model, 58.5 % accuracy of the VGG 16 model, and 54.9 % of the ensemble CNN model. By increasing the MRI input modalities, SurvNet becomes more accurate and achieves 76.5 % accuracy with four MRI modalities. Combining the segmented data, SurvNet achieved the highest accuracy of 82.4 %.

**Conclusions:**

The research results show that SurvNet achieves higher metrics such as accuracy and f1-score than the comparisons. Our research also proves that by using multiparametric MRI modalities, SurvNet is able to learn more image features and performs a better classification accuracy. We can conclude that SurvNet with the complete scenario, i.e., segmented data and four MRI modalities, achieved the best accuracy, showing the validity of segmentation information during the survival time prediction process.

## 1 Introduction

According to the World Health Organization (WHO) 2021 Central Nervous System (CNS) tumor classification criteria, glioblastoma (GBM) is the most aggressive form of gliomas and represents approximately 57 % of all gliomas and 48 % of all primary malignant brain tumors [1]. GBM is commonly grade IV glioma, results in a poor prognosis, which is

about 14 months of survival time after standard treatment such as surgical resection, radiotherapy, and chemotherapy, or 4 months of survival time without any treatments [2].

Radiomics is a non-invasive method to extract quantitative features from medical images that may not be apparent through traditional visual inspection. Automated image analytics with magnetic resonance imaging (MRI) can provide insights into the diagnosis of brain tumors and contribute to monitoring tumor change and therapy planning. Accurate identification of the overall survival (OS) time of patients with brain tumors is of profound importance in many clinical applications, such as surgical treatment planning, image-guided interventions, and the generation of radiotherapy maps. Given the very poor survival rate of patients, tools to stratify patients into those who could be targeted for clinical trials would be invaluable.

Machine learning algorithms, such as Support Vector Machine (SVM), Gradient Boosting Decision Tree (GBDT), and Random Forest, reported high classification performances on the Brain Tumor Segmentation (BraTS[3]) and The Cancer Genome Atlas (TCGA [4]) datasets. Liao et al. [5] compared 4 machine learning methods with radiomic features extracted from the fluid-attenuated inversion recovery (FLAIR) MRI scan and several specific gene expression features, reported a highest accuracy of 81 % with GBDT. Most of the top-ranked methods of survival prediction tasks in the BraTS dataset employed machine learning methods, such as Random Forest [6,7], Decision Tree [8], Linear Regression [9] and a Fully Connected Neural Network [10]. However, classical machine learning algorithms require manual feature extraction before training the model. This process is time-consuming, labor-intensive, and highly dependent on domain expertise.

Many studies have presented deep learning algorithms, based on convolutional neural networks (CNN), for processing and analysis of medical images of patients with brain tumors [11]. A 3D DenseNet121 was also used and obtained an accuracy of 55.2 % [12]. [13] introduce a CNN-based architecture and combined feature fusion method to achieve an accuracy of 65.57 %. Ahmed et al. [14] used a snapshot ensemble to choose and combine models trained from different epochs, reporting an accuracy of 72 %. Similarly, Ben Ahmed et al. [15] tested the snapshot ensemble model on MRI scans with different dimensionalities, reaching 60 % and 74 % accuracy with 2D and 3D MRI scans, respectively. The attention mechanism is also introduced into deep learning applications in medical imaging. Xu, Xuan et al. [16] proposed a DAAL(deep anchor attention learning) model based on an attention mechanism to achieve a classification accuracy of 70.29 %. Wenxia Wu et al. [17] proposed a multi-task Transformer encoder model to implement semi-supervised segmentation and survival analysis, and achieved 75.38 % on the survival prediction task [18]. introduced an encoder-based deep model in the BraTS dataset and achieved a 67.9 % accuracy.

The current methods mostly utilize merely a single modality of MRI images, while there is increasing evidence that quantitative analysis of radiographic features extracted from multiparametric MRI (mpMRI) scans leads to advanced image-based tumor phenotyping, which can be associated with predicting clinical outcomes [19]. Therefore, we explored a low-complexity architecture that extracts various deep features from diverse MRI modalities as well as the segmented data to improve classification accuracy. In this work, we address the application of convolutional neural network (CNN)-based deep learning methods for the overall survival prediction of patients with gliomas using mpMRI. The relevant code can be found at ‘https://github.com/OriginLyu/SurvNet’

Our contributions are:

1. We proposed SurvNet, a low-complexity CNN model to extract deep image features from MRI scans and classify the patients with GBM into long-survival and short-survival classes.
2. We performed several experiments including:
a. Evaluated SurvNet with the T1 MRI modality and compared the result of cross-validation with Inception V3, VGG16 and ensemble CNN models.
b. Examined the effect of increasing the number of input MRI modalities (from 1 to 4) on our proposed model and other comparisons.
c. Examined how adding segmented data to the input data might improve the models.

## 2 Methods

### 2.1 Data collection

TCGA, an open-source, open-access information resource created by the National Cancer Institute (NCI), has collections of MRI images, gene expression, and clinical information. A pre-processed MRI dataset, namely pre-operative_TCGA-GBM [20,21], was released based on The Cancer Genome Atlas Glioblastoma Multiforme Col-lection (TCGA-GBM) [22] dataset in 2017. Pre-operative_TCGA-GBM released segmentation labels and radiomics features for all pre-operative multimodal MRI (T1-weighted (T1), T2-weighted (T2), contrast-enhanced T1-weighted (T1ce), and FLAIR) of the multi-institutional glioma collections of TCGA.

The pre-operative_TCGA-GBM features standardized pre-processing, an essential procedure for MRI classification because of the diversity of different institutes that generate MRI scans. Pre-processing starts with a co-orientation to the left-posterior-superior coordinate system, then co-registering to the same anatomical template, followed by resampling to a uniform 1 mm^3^ voxel resolution. Skull-stripping is further applied to remove the skull in MR images, making the tumor region more conspicuous and mitigating potential facial reconstruction/recognition of the patients [23].

The pre-operative_TCGA-GBM dataset consists of 135 patients in total. Each patient has T1, T2, T1ce, FLAIR, and segmented MRI data. Out of these, we chose 119 cases with overall survival time (ranging from 5 to 2,126 days) and age (ranging from 17 to 84 years) to compose our subdata.

The 119 patients were divided into a training set with 104 individuals and a testing set with 17 patients. Both training and testing sets contained long-survival and short-survival cohorts. Clinically, the division criterion of 1 year is considered the beginning of long-term survival, which is, therefore, taken for the threshold of long-survival and short-survival. The training/testing set consists of 49/8 patients with short and 55/9 patients with long survival.

We produced 2D slices along the transverse axis from each of the original 3D MRI data. We chose a 2D slice with the largest area of the segmented brain tumor among the entire 2D slices using the maximum label voxel layer sampling method [24]. The input MR images (T1, T2, T1ce, and FLAIR) and the segmented data had a resolution of 240 × 240.

Apart from TCGA-GBM, we also used the BraTS dataset [2] to evaluate the performance of the proposed model. The BraTS dataset contains 236 samples with brain tumors (both GBM and low-grade glioma) and their MRI images. The BraTS dataset was divided into 201 and 35 samples for training and testing, respectively. The preprocessing of the BraTS dataset was the same as that of the TCGA-GBM dataset.

### 2.2 Architecture

**Fig. 1:**
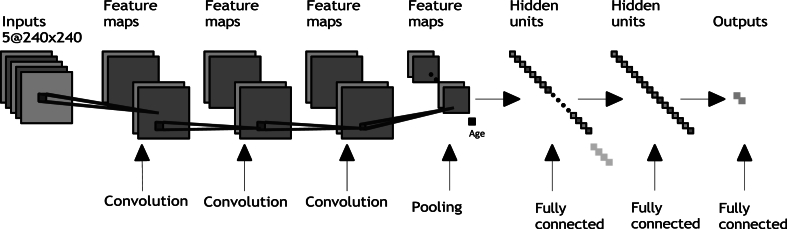
Architecture of SurvNet.

The proposed approach was based on VGG 16 but simplified at several levels. SurvNet was comprised of three convolutional blocks, one pooling layer for feature extraction, and three dense layers for classification, as shown in Fig. 1. Each convolutional block consisted of one convolutional layer and one activation layer with the Rectified Linear Unit (ReLU) function as the activation function. Apart from the deep features extracted from the MRI scans, the proposed model also made use of age information as one feature, combined with the deep features to predict the OS of patients.

Table 1 presents a more detailed view of the proposed architecture's parameters. The convolutional layer included a 3 × 3 kernel and 32 channels and uses the ‘padding = same’ method, which kept the image size the same after the convolutional layer by adding zero values into the edges of the image, followed by a ReLU activation layer. The pooling layer between the third convolutional block and the first fully connected layer used a max pooling method, with a 2 × 2 pooling window. As for the fully connected layers, the first and second layers contained 32 nodes and 16 nodes, respectively. The output layer was a fully connected layer with two nodes and a softmax activation function.

**Table 1:** Parameters of SurvNet.

Layer	Parameters
Conv block 1	3 × 3 × 32 stride = 1, padding = same activation = ReLU
Conv block 2	3 × 3 × 32 stride = 1, padding = same activation = ReLU
Conv block 3	3 × 3 × 32 stride = 1, padding = same activation = ReLU
Pooling	max pooling 2 × 2 stride = 2
FC 1	32 activation = ReLU
FC 2	16 activation = ReLU
Output	2 activation = softmax

Table 2 presents the optimized hyperparameters’ values, i.e., learning rate, decay rate, and the total number of epochs, concerning SurvNet (see Table 3).

**Table 2:** Hyperparameters of SurvNet.

Hyperparameter	Value
optimizer	stochastic gradient descent
learning rate	1 × 10^−5^
decay rate	2 × 10^−7^
epochs	80
batch size	16

**Table 3:** Model complexity.

Model/Metrics	Trainable parameters (M)	FLOPS (GFLOPS)
SurvNet	14.8	2.19
Inception V3	31.2	5.72
VGG 16	138	15.35
Ensemble CNN [14]	2.9	0.136

## 3 Results

### 3.1 Evaluation in T1 images

T1 MRI was taken as the base modality due to its widespread use in tumors. We compared the Inception V3 (both pre-trained Inception V3 on Imagenet dataset and an end-to-end Inception V3 model), a VGG16 model, and a CNN ensemble [14,15]. The pre-trained Inception V3 model used the Inception V3 model and pre-trained parameters based

on the Imagenet dataset. Three dense layers were added at the end of the whole pre-trained Inception V3 model as a classifier to be trained on our dataset. The end-to-end Inception V3 model trained the weights in all of its layers on our dataset. The frozen Inception V3 model froze Inception architecture and tuned the classifier only. The Inception V3 models, the VGG16 model, and the CNN ensemble CNN employed the T1 MRI modality as input.

**We tuned the hyperparameters of SurvNet by the grid search of the learning rate, decay rate, and batch size. We selected the feasible range of hyperparameters and then chose the best hyperparameters according to the training loss and train accuracy. The number of epochs was chosen when the training loss converged.** The comparison models were also optimized using the same procedure.

We took a 3-fold cross-validation to evaluate the performance of all models mentioned earlier, including the proposed one. The dataset was divided randomly into three parts, with two parts used for training and one part for testing. The hyperparameters of cross-validation were set as the optimized ones in Table 2. Let *a*_*i*,*j*_ be the accuracy of a particular model at the *i*th round (1 ≤ *i* ≤ 3) and *j*th epoch (1 ≤ *j* ≤ 80). We then computed the mean accuracy as follows:

(1) $$\begin{array}{c} \mu_{i}=\frac{1}{3}\sum_{i=1}^{3}a_{ij} \end{array}$$

and standard deviation according to:

(2) $$\begin{array}{c} \sigma_{j}=\sqrt{\frac{1}{3}\sum_{i=1}^{3}{\left(a_{ij}-\mu_{j}\right)}^{2}} \end{array}$$

**Fig. 2:**
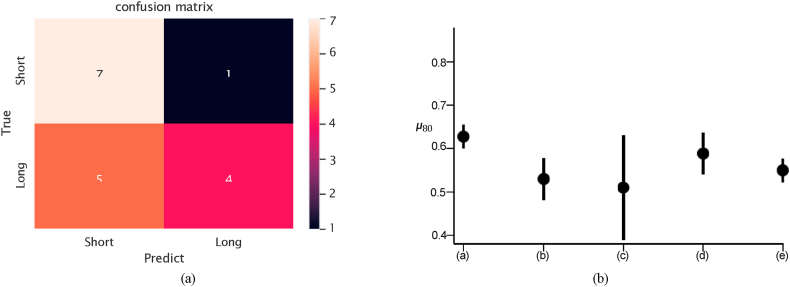
(a) Confusion matrix on the testing set (T1 modality only), (b) *μ*_80_ and δ_80_of: (a) SurvNet with 1 MRI modality, (b) Inception with 1 MRI modality, frozen, (c) Inception with 1 MRI modality, end-to-end, (d) VGG16 with 1 MRI modality, (e) Ensemble CNN with 1 MRI modality.

Both measures were computed for each model, as depicted in Fig. 2 (b), which illustrates the ***μ***_80_ value of each model, with lines reflecting the standard deviation. SurvNet achieved the best accuracy value of 62.7 %.

The confusion matrix is shown in Fig. 2 (a). We considered the accuracy, precision, recall, and F1-score as the evaluating measures over the 3-fold cross-validation to evaluate the classification ability of SurvNet. Table 4 presents the results. SurvNet achieved an accuracy of 62.7 %, higher than Inception V3 models, VGG 16 model and the ensemble CNN approach. However, the F1-score of SurvNet was lower than that of the VGG 16 and ensemble CNN models, for our recall is relatively low. As for the performance on the BraTS dataset, SurvNet achieved an accuracy of 68.6 %,

**Table 4:** Experimental results considering the T1 modality.

Dataset	Model/Metrics	Accuracy Precision Recall F1-score			
	SurvNet (1 modality)	0.627	0.783	0.407	0.535
	Inception V3 (pre-trained, 1 modality)	0.529	0.552	0.556	0.553
TCGA-GBM	Inception V3 (end-to-end, 1 modality) VGG 16	0.510	0.514	0.481	0.489
		0.588	0.524	0.630	0.570
	Ensemble CNN (1 modality) [14]	0.549	0.576	0.556	0.562
	Ensemble CNN (1 modality) [15]	0.609	0.586	**0.739**	**0.654**
	SurvNet (1 modality)	0.686	0.636	0.824	0.718
	Inception V3 (pre-trained, 1 modality)	0.514	0.5	0.647	0.564
BraTS	Inception V3 (end-to-end, 1 modality) VGG 16	0.657	0.6	0.882	0.714
		0.543	0.52	0.765	0.619
	Ensemble CNN (1 modality) [14]	0.588	0.625	0.556	0.588
	Ensemble CNN (1 modality) [15]	0.67	0.7	0.65	0.674

which was higher than the Inception V3, VGG16 and ensemble CNN model. With the introduction of more training samples, the performance of each model improved.

As for the computational load, the Inception V3, VGG16, and the ensemble CNN models contained 31.2 M, 138 M, and 2.9 M trainable parameters, respectively, while SurvNet contained 14.8 M trainable parameters. The ensemble CNN model took about 87 % of the training time of SurvNet. However, the inference time in the ensemble CNN model was almost fourfold higher than that in SurvNet, for the ensemble method had to compute the output of all its models. SurvNet with the T1 MRI modality was quicker than the ensemble CNN model.

### 3.2 Evaluation in different modalities

To examine the effect of increasing the number of input MRI modalities, we measured the performance of SurvNet with different input combinations of MRI modalities. We used T1 in our initial experiment given the good performance in previous experiments described above, because it does not require a contrast agent. Other than T1, we included FLAIR, as it can show abnormalities clearly [5]. Additionally, we set T2 as the third priority, for this modality is part of almost all brain tumor MRI protocols [25]. **To avoid that other modalities may be more contributing than T1, we also tested the proposed model with every single modality and the SurvNet with other modalities has the same or similar performance as it with T1.**

We considered four scenarios: (i) T1 only, (ii) T1 and FLAIR, (iii) T1, FLAIR, and T2, and (iv) T1, FLAIR, T2, and T1ce. The hyperparameters of SurvNet with different combinations of input MRI modalities are set as presented in Table 2. The 3-fold cross-validation is applied to all models.

**Fig. 3:**
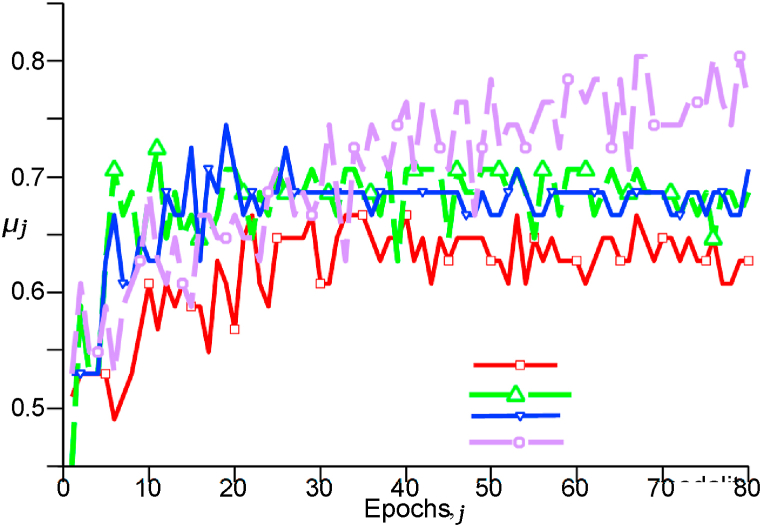
Evaluation of ***μ***_*j*_ considering different MRI modalities in SurvNet.

**Fig. 4:**
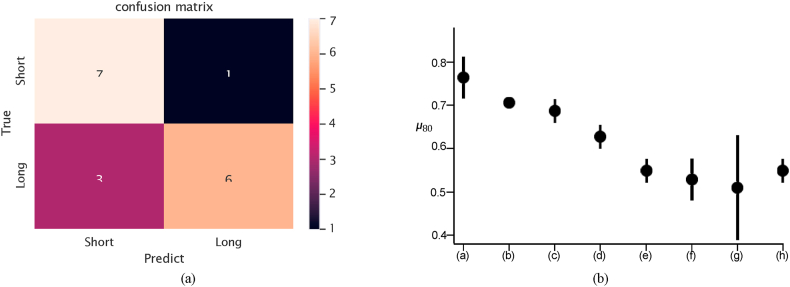
(a) Confusion matrix on the testing set (4 modalities), (b) *μ*_80_ and δ_80_ of: (a) SurvNet with 4 MRI modalities, (b) SurvNet with 3 MRI modalities, (c) SurvNet with 2 MRI modalities, (d) SurvNet with 1 MRI modality, (e) Inception with 3 MRI modalities, end-to-end, (f) Inception with 1 MRI modality, frozen, (g) Inception with 1 MRI modality, end-to-end, (h) Ensemble CNN with 1 MRI modality.

Fig. 3 shows the mean accuracy at epoch *i* of SurvNet, denoted here by ***μ***_*j*_. One can observe that SurvNet reached the highest accuracy with all MRI modalities. We compared the *μ*_0_ and ***δ*** δ_80_ of each model, as shown in Fig. 4 (b). The points indicate each model's *μ*_80_, and the lines reflect the ***σ*** δ_80_ values. As a result, the *μ*_80_ of SurvNet increased from one modality to four, which did not necessarily happen with the other architectures. The end-to-end Inception, for instance, had a considerably high standard deviation.

Table 5 presents the outcomes of the experimental results concerning different modalities. As we increased the number of MRI modalities, SurvNet improved its generalization ability in all measures, which is presented in Fig. 4

**Table 5:** Performance of the models with various input modalities.

Dataset	Model/Metrics	Accuracy	Precision	Recall	F1-score	AUC
	SurvNet with 4 MRI modalities	0.765	0.827	0.704	0.760	0.875
	SurvNet with with 3 MRI modalities	0.706	0.833	0.556	0.667	0.764
TCGA-GBM	SurvNet with with 2 MRI modalities	0.686	0.794	0.556	0.653	0.736
	SurvNet with with 1 MRI modality	0.627	0.783	0.407	0.535	0.722
	Inception with 3 MRI modalities	0.549	0.545	0.704	0.607	0.646
	Ensemble CNN (1 modality) [15]	0.609	0.586	0.739	0.654	–
	SurvNet with 4 MRI modalities	0.771	0.8	0.766	0.750	0.788
	SurvNet with with 3 MRI modalities	0.714	0.889	0.471	0.615	0.778
BraTS	SurvNet with with 2 MRI modalities	0.714	0.889	0.471	0.615	0.708
	SurvNet with with 1 MRI modality	0.686	0.636	0.824	0.718	0.690
	Inception with 3 MRI modalities	0.686	0.636	0.824	0.718	0.690
	Ensemble CNN (1 modality) [15]	0.67	0.7	0.65	0.674	0.67

(a). SurvNet achieved the overall best result with all modalities, with 76.5 % accuracy and 0.760 F1-score on pre-operaitve_TCGA-GBM and 77.1 % accuracy and 0.778 F1-score on BraTS. The receiver operative characteristic (ROC) of SurvNet with one to four modalities is depicted in Fig. 5, yielding an area under the curve (AUC) value presented in Table 5. Fig. 5 represents the true positive rate (y-axis) and false positive rate (x-axis) for each input combination at various classification thresholds. The optimal thresholds for each model are selected to achieve maximal true positive and minimal false positive rates. As shown in Fig. 5, SurvNet performance significantly improved as the number of

**Fig. 5:**
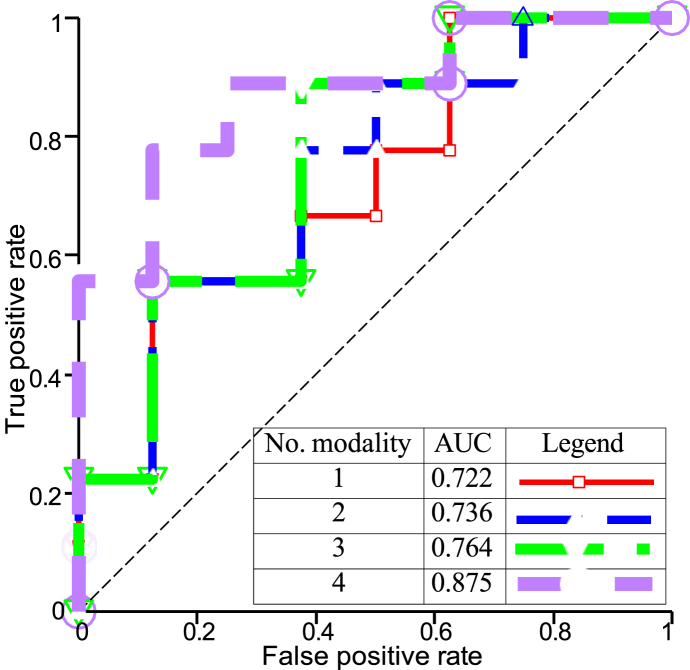
ROC analysis of SurvNet with various input MRI modalities.

MRI modalities increased as shown by the AUCs. Therefore, SurvNet with all MRI modalities was chosen because it presents higher accuracy. SurvNet started with a convolutional layer that outputs 32 feature maps, therefore, SurvNet's parameters stayed constant regardless of the number of input MRI modalities (see Table 6

**Table 6:** Performance of the models with segmented data and different input modalities.

Dataset	Model/Metrics	Accuracy	Precision	Recall	F1-score	AUC
	SurvNet with segmented data and 4 modalities	0.824	0.889	0.778	0.822	0.931
	SurvNet with segmented data and 3 modalities	0.765	0.804	0.740	0.769	0.847
TCGA-GBM	SurvNet with segmented data and 2 modalities	0.725	0.813	0.630	0.708	0.736
	SurvNet with segmented data and 1 modality	0.687	0.822	0.519	0.635	0.750
	SurvNet with segmented data	0.647	0.756	0.519	0.603	0.708
	SurvNet with segmented data and 4 modalities	0.8	0.813	0.765	0.788	0.853
	SurvNet with segmented data and 3 modalities	0.743	0.9	0.529	0.667	0.737
BraTS	SurvNet with segmented data and 2 modalities	0.714	0.733	0.647	0.688	0.712
	SurvNet with segmented data and 1 modality	0.657	0.667	0.588	0.625	0.655
	SurvNet with segmented data	0.629	0.625	0.588	0.606	0.627

### 3.3 Evaluation with different modalities and segmented data

Besides the four MRI modalities, the pre-operative_TCGA-GBM dataset provided the segmented data, where the brain tumor sections were segmented from the original MRI using all the T1, T2, FLAIR and T1ce MRI modalities [20]. The segmented data is cropped MRI images, including the whole tumor (WT), the tumor core (TC), the enhancing part of the tumor core (ET), the non-enhancing part of the tumor core (NET), and the peritumoral edema (ED). To further evaluate the effect of segmented data, we conducted a series of experiments based on the combination of input data as follows: segmented data only; segmented data and T1; segmented data, T1 and FLAIR; segmented data, T1, FLAIR, and T2; segmented data, T1, FLAIR, T2 and T1ce. These experiments’ hyperparameters were the same as presented in Table 2.

**Fig. 6:**
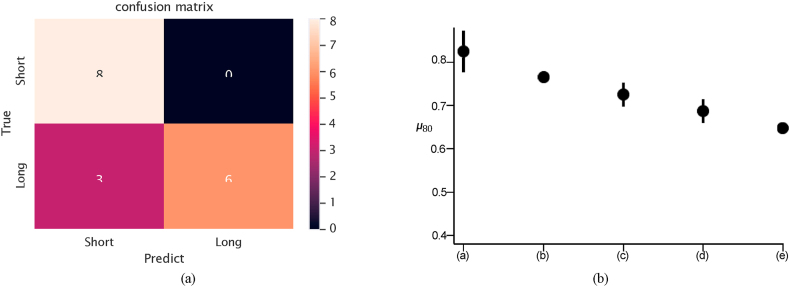
(a) Confusion matrix on the testing set (4 modalities and seg), (b) *μ*_80_ and δ_80_ of SurvNet with various inputs: (a) Segmented data and 4 MRI modalities, (b) Segmented data and 3 MRI modalities, (c) Segmented data and 2 MRI modalities, (d) Segmented data and 1 MRI modality, (e) Segmented data only.

**Fig. 7:**
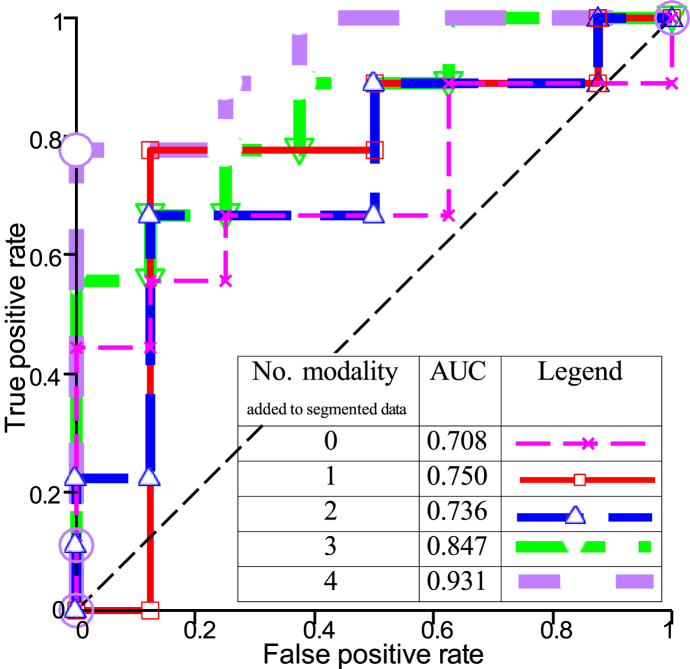
ROC analysis of SurvNet with different input MRI modalities and segmented data.

Fig. 6 (b) presents the results of the 3-fold cross-validation. Again, the ***μ***_80_ values of SurvNet increased as the number of input modalities grew. SurvNet using segmented data and four MRI modalities achieved the best performance of 82.4 % accuracy and 0.822 F1-score on pre-operative_TCGA-GBM and 80 % accuracy and 0.788 F1-score on BraTS. Additionally, Fig. 7 displays the ROC curves concerning SurvNet with five input image combinations. The increased number of input MRI modalities results in a larger area under the ROC. SurvNet with the segmented data and four MRI modalities reached the highest AUC.

**Fig. 8:**
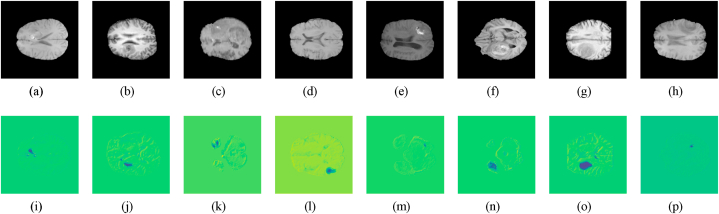
**Feature map of SurvNet**, (a)–(h) are the T1 images, and (i)–(p) are the feature maps.

**Fig. 9:**
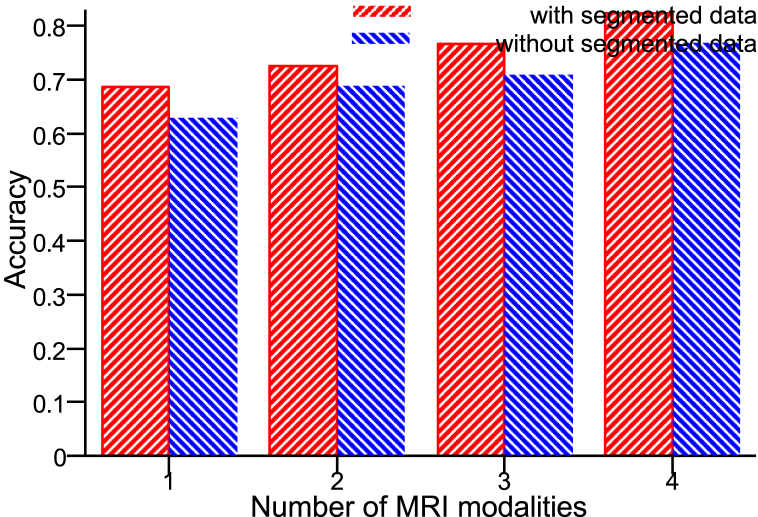
Evaluation of SurvNet: with and without the segmented data.

Fig. 9 depicts a comparison of the ***μ***_80_ values concerning SurvNet with different MRI modalities and segmented data. We can conclude that SurvNet with the complete scenario, i.e., segmented data and four MRI modalities, achieved the best accuracy, showing the validity of segmentation information during the survival time prediction process. We visualized the feature maps of the last convolutional layer of SurvNet, as shown in Fig. 8. The feature maps present the highly

concatenated features inside and around the region of the brain tumor. The lightweight architecture can benefit from the high contrast between different tissues from MRI, therefore, learning the features efficiently and accurately.

## 4 Discussion

In this paper, we proposed SurvNet, a low-complexity CNN, for predicting the overall survival time of patients affected by GBM. The pre-operative_TCGA-GBM dataset with 119 individuals was divided into long-survival and short-survival cohorts via the threshold of one year and into training and testing sets with a ratio of 85:15. The BraTS with 236 samples are also used to validate the performance of SurvNet. Firstly, we evaluated SurvNet with the T1 modality and compared it with Inception V3, VGG16 and ensemble CNN models. Secondly, we examined the effect of increasing input MRI modalities on our proposed model. Finally, we examined how adding segmented data to the input data might improve the models.

SurvNet with T1 MRI modality achieved a 62.7 % accuracy, compared with 52.9 % accuracy of the Inception V3 model, 58.5 % accuracy of the VGG 16 model, and 54.9 % of the ensemble CNN model. By increasing the MRI input modalities, SurvNet becomes more accurate and achieves 76.5 % accuracy with four MRI modalities. Combining the segmented data, SurvNet achieved the highest accuracy of 82.4 % on pre-operative_TCGA-GBM and 80 % on BraTS. The segmented data appear as helpful for OS prediction as a single whole-brain MRI modality. We also calculated other metrics, such as precision, recall, f1-score, and AUC, which show the same tendency as accuracy when increasing the number of input MRI modalities. To the best of our knowledge, our results are state-of-the-art on the pre-operative_TCGA-GBM dataset. As for the computational load, SurvNet compared favorably to the alternative models assessed.

The proposed model has advantages in the model complexity. The feature maps present the highly concatenated features inside and around the region of the brain tumor. The improvement that the lightweight architecture brings is from the high contrast between different tissues and significant features of MRI. Low-complexity models can learn from these clear features more efficiently and make accurate predictions. Besides, the low-complexity model can avoid the risk of overfitting in the small dataset.

In the clinical application, SurvNet can improve risk stratification by providing accurate survival predictions, to categorize patients into different risk groups more accurately. With a more accurate prognosis, SurvNet can help in choosing the most appropriate treatment plan. **For instance, patients with a predicted survival of less than 1 year might more benefit from having no treatment, while those with higher survival prediction might benefit from intensification. The clinical practice is complicated and a single model is not decisive for the treatment plan, but we still hope that this kind of research can help better understand and evaluate the patient's prognosis.** In the meanwhile, SurvNet also meets some challenges and limitations. The best performance of SurvNet requires the input of all MRI modalities and segmented data, which might not be available in some clinical settings. The small dataset size may have a potential bias against a large number of clinical data, therefore limiting the generalization of the model.

## 5 Conclusion

We have built an accurate tool to stratify patients into those with poor OS who could be targeted for clinical trials. Given the very poor survival rate of patients, this has a large potential value.

Through the incorporation of diverse MRI modalities as inputs, we facilitate deep feature extraction at various anatomical sites, thereby augmenting the precision of predictive modeling. Four MRI modalities, T1, T2, FLAIR and T1ce as well as the segmented data are tested as various input combinations into the low-complexity CNN-based model we proposed. In the meanwhile, we compared SurvNet with Inception V3, VGG 16 and ensemble CNN models. Three series of experiments are performed: 1. compared SurvNet with T1 MRI modality with Inception V3, VGG 16 and ensemble CNN models with T1 MRI modality; 2. compared SurvNet by increasing the number of input MRI modalities; 3. tested SurvNet by adding segmented data with MRI modalities. In the three-fold cross-validation, SurvNet with T1 modality achieved 62.7 % accuracy, which is better than the comparisons. Inputting more MRI modalities and including segmented data, SurvNet achieved more accurate and stable performance in the three-fold cross-validation, where SurvNet with the input of 4 MRI modalities and segmented data achieved 82.4 % accuracy. We can conclude that SurvNet with the complete scenario, i.e., segmented data and four MRI modalities, achieved the best accuracy, showing the validity of segmentation information in the task of OS prediction. Apart from the pre-operative_TCGA-GBM, we also tested SurvNet on the BraTS dataset, where SurvNet with the input of 4 MRI modalities and segmented data achieved the highest 80 % accuracy.

One limitation of our research is the small dataset, with only 119 patients. Although this is not small compared to other GBM studies, large datasets are required to evaluate the performance further. In particular, whilst TCGA data is obtained from multiple sites, further validation using data from an entirely different dataset might prove the generalisability. Another limitation is that other MRI modalities that might be helpful for the classification task, such as diffusion-weighted imaging or advanced imaging have not been included. Apart from the GBM, many other types of brain tumors have a better prognosis than GBM, which is not suitable for the two-way classification task.

## Data availability statement

The authors do not have permission to share data. The datasets used in this study are publicly available and can be accessed from the following sources. This paper utilizes the clinical data from TCGA-GBM and pre-processed MRI data from pre-operative_TCGA-GBM. The two datasets can be downloaded online from:

• TCGA-GBM: Scarpace, L., Mikkelsen, T., Cha, S., Rao, S., Tekchandani, S., Gutman, D., Saltz, J. H., Erickson,

B. J., Pedano, N., Flanders, A. E., Barnholtz-Sloan, J., Ostrom, Q., Barboriak, D., & Pierce, L. J. (2016). The Cancer Genome Atlas Glioblastoma Multiforme Collection (TCGA-GBM) (Version 4) [Data set]. The Cancer Imaging Archive. https://doi.org/10.7937/K9/TCIA.2016.RNYFUYE9

• pre-operative_TCGA-GBM: Bakas S, Akbari H, Sotiras A, Bilello M, Rozycki M, Kirby J, Freymann J, Farahani K, Davatzikos C. (2017). Segmentation Labels for the Pre-operative Scans of the TCGA-GBM collection [Data set]. The Cancer Imaging Archive. DOI: 10.7937/K9/TCIA.2017. KLXWJJ1Q
• BraTS: Menze, Bjoern H. and Jakab, Andras and Bauer, Stefan and Kalpathy-Cramer, Jayashree and Farahani, Keyvan and Kirby, Justin and Burren, Yvonne and Porz, Nicole and Slotboom, Johannes and Wiest, Roland and Lanczi, Laura and Gerstner, Elizabeth and Weber, Marc-André and Arbel, Tal and Avants, Brian B. and Ayache, Nicholas and Buendia, Patricia and Collins, D. Louis and Cordier, Nicolas and Corso, Jason J. and Criminisi, Antonio and Das, Talisha and Delingette, Hervé and Demiralp, Çetin and Durst, Christopher R. and Dojat, Michel and Doyle, Scott and Festa, Joshua and Forbes, Fiona and Geremia, Elena and Glocker, Ben and Golland, Polina and Guo, Xiahai and Hamamci, Ali and Iftekharuddin, Khan M. and Jena, Rajiv and John, Nigel M. and Konukoglu, Ender and Lashkari, Danial and Mariz, Jorge A. and Meier, Raphael and Pereira, Sérgio and Precup, Doina and Price, Stephen J. and Raviv, Tamir R. and Reza, S. M. Masud and Ryan, Michael and Sarikaya, Duygu and Schwartz, Lawrence and Shin, Hoo-Chul and Shotton, Jamie and Silva, Claudia A. and Sousa, Nuno and Subbanna, N. Karthik and Szekely, Gabor and Taylor, Thomas J. and Thomas, Owen M. and Tustison, Nicholas J. and Unal, Gozde and Vasseur, Francis and Wintermark, Max and Ye, Dexin H. and Zhao, Ling and Zhao, Binsheng and Zikic, Darko and Prastawa, Marcel and Reyes, Mauricio and Van Leemput, Koen,

“The Multimodal Brain Tumor Image Segmentation Benchmark (BRATS),” IEEE Transactions on Medical Imaging, vol. 34, no. 10, pp. 1993–2024, Oct. 2015. https://doi.org/10.1109/TMI.2014.2377694. Epub 2014 Dec 4. PMID:

25494501; PMCID: PMC4833122

## Declarations

All authors have made substantial contributions to the work, including the conception and design of the work, the acquisition, analysis, and interpretation of data, drafting the work and revising it critically for important intellectual content, and final approval of the version to be published.

None of the authors of this paper has a financial or personal relationship with other people or organizations that could inappropriately influence or bias the content of the paper.

## CRediT authorship contribution statement

**Qiyuan Lyu:** Writing – original draft, Validation, Software, Methodology, Formal analysis, Data curation. **Mario Parreno-Centeno:** Writing – review & editing, Methodology, Data curation. **João Paulo Papa:** Writing – review & editing, Investigation. **Esin Öztürk-Isik:** Writing – review & editing. **Thomas C. Booth:** Writing – review & editing, Investigation. **Fumie Costen:** Writing – review & editing, Supervision, Funding acquisition, Conceptualization.

## Declaration of competing interest

The authors declare that they have no known competing financial interests or personal relationships that could have appeared to influence the work reported in this paper.
